# Supplementary material for: Alterations in innate immune responses of patients with chronic rhinosinusitis related to cystic fibrosis
Source: PLoS One. 2022 May 6;17(5):e0267986. doi: 10.1371/journal.pone.0267986 (PMC9075614; doi:10.1371/journal.pone.0267986)
Supplement: S1 Table — (DOCX) [file pone.0267986.s001.docx]

**S1 Table. Phagocytosis of *Saccharomyces cerevisiae* cells by pathogen-associated molecular pattern receptors in the peripheral leukocytes of children; values are expressed as median.**

| **Groups** | **Cell/yeasts** | **Monocyte** | | | **Neutrophil** | | |
| --- | --- | --- | --- | --- | --- | --- | --- |
|  |  | *%CIP* | *Yeasts by cell* | *PhI* | *%CIP* | *Yeasts by cell* | *PhI* |
| **Control** | ***1/5*** | 14.3 | 1.4 | 18.3 | 14.8 | 1.4 | 18.8 |
|  | ***1/20*** | 17.5 | 1.4 | 20.8 | 26.0 | 1.4 | 20.8 |
| **Cystic fibrosis** | ***1/5*** | 13.5 | 1.5 | 19.5 | 1.5* | 1.0* | 1.5* |
|  | ***1/20*** | 37.0 | 1.6 | 59.0 | 1.0 | 1.0 | 1.0 |
| **CF+CRS**  **with NP** | ***1/5*** | 6.5 | 1.7 | 12.7 | 1.5* | 1.0* | 2.0* |
|  | ***1/20*** | 8.7 | 1.8 | 14.1 | 0.5 | 1.3 | 1.5 |
| **CF+CRS**  **without NP** | ***1/5*** | 6.5 | 1.3 | 13.0 | 3.7 | 1.9 | 5.0 |
|  | ***1/20*** | 21.7 | 1.6 | 36.0 | 4.5 | 1.5 | 6.4 |

CF = Cystic fibrosis; CRS = chronic rhinosinusitis; NP = nasal polyps; %CIP = percentage of cells involved in phagocytosis; PhI = phagocytic index. *Values are different from their respective control (p <0,05)
