# Supplementary material for: Alterations in innate immune responses of patients with chronic rhinosinusitis related to cystic fibrosis
Source: PLoS One. 2022 May 6;17(5):e0267986. doi: 10.1371/journal.pone.0267986 (PMC9075614; doi:10.1371/journal.pone.0267986)
Supplement: S2 Table — (DOCX) [file pone.0267986.s002.docx]

**S2 Table.** Phagocytosis of *Saccharomyces cerevisiae* by opsonin receptors in the peripheral leukocytes of children; the values are expressed as median values.

| **Groups** | **Cell/yeasts** | **Monocyte** | | | **Neutrophil** | | |
| --- | --- | --- | --- | --- | --- | --- | --- |
|  |  | *%CIP* | *Yeasts by cell* | *PhI* | *%CIP* | *Yeasts by cell* | *PhI* |
| **Control** | ***1/5*** | 53.0 | 2.0 | 102.5 | 53.0 | 2.0 | 102.5 |
|  | ***1/20*** | 71.5 | 2.6 | 198.0 | 71.5 | 2.6 | 198.0 |
| **Cystic fibrosis** | ***1/5*** | 51.3 | 1.5 | 24.5 | 40.6 | 1.4 | 61.1 |
|  | ***1/20*** | 70.8 | 1.9 | 152.5 | 75.2 | 1.7* | 99.2* |
| **CF+CRS**  **with NP** | ***1/5*** | 54.5 | 1.7 | 55.8* | 38.1 | 1.4 | 52.5 |
|  | ***1/20*** | 77.2 | 2.1 | 125.8 | 66.5 | 1.7* | 97.1* |
| **CF+CRS**  **without NP** | ***1/5*** | 50.5 | 1.4 | 19.6* | 36.8* | 1.3 | 34.6 |
|  | ***1/20*** | 79.2 | 2.1 | 154.0 | 71.6 | 1.6* | 106.7 |

CF = Cystic fibrosis; CRS = chronic rhinosinusitis; NP = nasal polyps; %CIP = percentage of cells involved in phagocytosis; PhI = phagocytic index. *Values different from their respective control (p < 0,05)
