# Supplementary material for: Alterations in innate immune responses of patients with chronic rhinosinusitis related to cystic fibrosis
Source: PLoS One. 2022 May 6;17(5):e0267986. doi: 10.1371/journal.pone.0267986 (PMC9075614; doi:10.1371/journal.pone.0267986)
Supplement: S1 File — (PDF) [file pone.0267986.s003.pdf]

NEUTROPHILS -phagocytosis PAMP			
% neutrophils 1/5			
Control	Cystic fibrosis	CF+CRSwNP	CF+CRSnNP
2	1,5	1	0,5
51,5	11,5	1	5,5
7	1	2	3
7	1,5	0	4,3
4,5	1,5	14,6	
7		5,6	
14,5			
14			
15			
17			
21			
31			
36			
40			
Mean yeasts 1/5			
Control	Cystic fibrosis	CF+CRSwNP	CF+CRSnNP
1	1	1	3
1,5	1,1	2	1,3
1,5	1	1	2,1
1,6	1	0,1	1,7
1,4	1	1	
1,4		1,3	
1,3			
1			
1,1			
1,2			
1,4			
1,6			
1,7			
1,9			
PI 1/5			
Control	Cystic fibrosis	CF+CRSwNP	CF+CRSnNP
10,5	1,5	1	1,5
11,5	13	2	7
6,5	1	2	4,3
9,5	1,5	0,1	5,6
18,5	1,5	14,6	
18		6,9	
19			
24			

30,5			
38			
38,5			
40			

NEUTROPHILS -phagocytosis PAMP			
% neutrophils 1/20			
Control	Cystic fibrosis	CF+CRSwNP	CF+CRSnNP
1,5	0,5	0,5	3,5
	0,5	1,5	5
8,5	32,5	0	4,3
8	1	0,5	4,6
6,5	3,5	0	
8,5		2,1	
6		1	
26			
27			
29			
39			
43			
46,5			
49			
Mean yeasts 1/20			
Control	Cystic fibrosis	CF+CRSwNP	CF+CRSnNP
1	1	1	1,7
1,4	1	1	1,3
1,8	1,2	3	1,5
1,4	1	0,1	1,4
1,2	1,4	1,5	
2,6		1,5	
1			
1			
1			
1,4			
1,9			
1,9			
1,9			
PI 1/20			
Control	Cystic fibrosis	CF+CRSwNP	CF+CRSnNP
1,5	0,5	0,5	6
11,5	0,5	1,5	6,5

14	38,5	1,5	6,3
9	1	0,1	6,4
10	5	3,2	
15,5		1,5	
26			
28,5			
33			
46			
55			
58			

Neutrophil Opsonins			
% 1/5			
Control	Cystic fibrosis	CF+CRSwNP	CF+CRSnNP
49,5	47	50	38,5
86	31	26,2	48
52	5,5	68,8	50
97	79	22	34,5
54	40,6	61,7	35
50,5		25	25,8
96,5			
41			
46			
49			
51,5			
60			
69			
75			
mean yeasts 1/5			
Control	Cystic fibrosis	CF+CRSwNP	CF+CRSnNP
1,2	1,3	1,4	1,3
1,9	1,2	1,4	1,3
2	1,4	1,6	1,3
4,4	1,8	1,1	1,7
2	1,4	1,2	1,3
2			1,4
3,4			1,2
1,1			
1,1			
1,3			
1,6			
2,1			
2,3			

2,4			
IF 1/5			
Control	Cystic fibrosis	CF+CRSwNP	CF+CRSnNP
58,7	61,1	52,5	34,6
106,5	61,5	66	63,3
106	36	81	32,9
102,5	7,5	39,5	127,1
74	142	43	28
76			85,7
77			30
91			
108			
111			
124			

Neutrophils Opsonins			
% 1/20			
Control	Cystic fibrosis	CF+CRSwNP	CF+CRSnNP
81,5	66,7	44,9	71,6
69	76	46	65,4
50,5	60	66,8	95,7
97	98,1	98,9	52,5
53	75,2	66,5	90,7
56		81,5	
88,5		64,7	
67			
68			
70			
73			
83			
86			
88			
Mean yeasts 1/20			
Control	Cystic fibrosis	CF+CRSwNP	CF+CRSnNP
2	1,7	1,6	1,4
1,9	1,7	1,7	1,6
2,1	1,5	1,7	2,6
3,3	2,8	1,6	1,3
2	1,9	2	1,9
2,1		1,4	
4,1			

2,4			
2,5			
2,6			
3,2			
3,6			
4			
4,8			
IF 1/20			
Control	Cystic fibrosis	CF+CRSwNP	CF+CRSnNP
165	111,4	71,3	100,5
131,5	1,5	77	106,7
108,5	128,5	112,2	250,7
319,5	87	106	69
105		165,5	170
115,5		88,2	
363			
192			
197			
199			
242			
298			
301			
342			
