# Supplementary material for: Alterations in innate immune responses of patients with chronic rhinosinusitis related to cystic fibrosis
Source: PLoS One. 2022 May 6;17(5):e0267986. doi: 10.1371/journal.pone.0267986 (PMC9075614; doi:10.1371/journal.pone.0267986)
Supplement: S2 File — (PDF) [file pone.0267986.s004.pdf]

MONOCYTES phagocytosis PAMPs			
% 1/5			
Control	Cystic fibrosis	CF+CRS with NP	CF+CRS without NP
2	3	0,5	17,5
5,7	31,6	6,5	3
7	6,5	2	7,92
7	13,5	34,8	2,5
4,5	36,3	9,1	7,7
7			5,3
14,5			
14			
15			
17			
21			
31			
36			
40			
Mean yeasts 1/5			
Control	Cystic fibrosis	CF+CRSwNP	CF+CRSnNP
1	1,7	1,5	1
1,5	1,5	1,7	2,2
1,5	1,4	2,3	1
1,6	1,4	1,2	1,5
1,4	1,6	1,7	1,3
1,4			
1,3			
1			
1,1			
1,2			
1,4			
1,6			
1,7			
1,9			
PI 1/5			
Control	Cystic fibrosis	CF+CRSwNP	CF+CRSnNP
2	5	0,5	25,5
8,5	46,6	14	5
10,5	9	2	18,32
11,5	19,5	52,2	3
6,5	59	11,4	13
9,5		16	
18,5			

18			
19			
24			
30,5			
38			
38,5			
40			

Monocytes PAMPS			
% 1/20			
Control	Cystic fibrosis	CF+CRS with NP	CF+CRS without NP
6	1	38	35,5
3,5	53,4	6	15,5
8,5	5,5	10,3	4,1
8	38	5,5	31,5
6,5	37	20,5	21,7
8,5		7	
6			
26			
27			
29			
39			
43			
46,5			
49			
Mean yeasts 1/20			
Control	Cystic fibrosis	CF+CRSwNP	CF+CRSnNP
1,6	1	2,2	1,6
1,4	1,6	1,9	1,5
1,4	1,2	1,6	1,6
1,8	1,6	1,3	1,8
1,4	1,6	1,9	1,6
1,2		1,6	
2,6			
1			
1			
1			
1,4			
1,9			
1,9			
1,9			
PI 1/20			

Control	Cystic fibrosis	CF+CRSwNP	CF+CRSnNP
9,5	1	82,4	57
5	84,5	11,5	22,5
11,5	6,5	16,7	6,5
14	59	7	58
9	60	38,5	36
10		11,1	
15,5			
26			
28,5			
33			
46			
55			
58			
74			

MONOCYTES OPSONINS			
% 1/5			
Control	Cystic fibrosis	CF+CRSwNP	CF+CRSnNP
46,6	39	50,5	27,6
89,1	51,5	59,7	33
52	28,6	21,8	53,9
97	86	82,2	50,5
54	51,3	22,2	59
50,5		58,5	
96,5			
41			
46			
49			
51,5			
60			
69			
75			
Mean yeasts 1/5			
Control	Cystic fibrosis	CF+CRSwNP	CF+CRSnNP
1,5	1,5	1,7	1,3
2	1,4	1,6	1,7
2	1,4	1,3	1,3
2	2	2,2	1,4
2	1,6	1,3	1,8
3,4		1,8	
1,1			
1,1			

1,3			
1,6			
2,1			
2,3			
2,4			
PI 1/5			
Control	Cystic fibrosis	CF+CRSwNP	CF+CRSnNP
70,1	24,5	1	57
106,5	0,5	84,5	22,5
106	5,5	6,5	6,5
102,5	98	59	58
74	34,7	60	29
76		52,5	82,4
77			11,5
91			16,7
108			7
111			38,5
124			11,1

MONOCYTE OPSONINS			
% 1/20			
Control	Cystic fibrosis	CF+CRSwNP	CF+CRSnNP
87	46,5	72,3	79,2
69	78,5	50,2	76
50,5	63	93	83,7
97	95,3	82	55,5
53	70,8	86,8	98
56		20,6	
88,5			
67			
68			
70			
73			
83			
86			
88			
Mean yeasts 1/20			
Control	Cystic fibrosis	CF+CRSwNP	CF+CRSnNP
2,1	1,6	2,1	1,9
1,6	1,9	1,6	2,1
2,1	1,8	2,2	2,5

3,3	2,9	2	1,6
2	2,1	2,3	2,8
2,1		1,3	
4,1			
2,4			
2,5			
2,6			
3,2			
3,6			
4			
4,8			
PI 1/20			
Control	Cystic fibrosis	CF+CRSwNP	CF+CRSnNP
184	76	128	150,8
113	152,5	99	157,5
108,5	115,5	167,2	206,1
319,5	272,6	123,5	89,5
105	154,2	113,3	270,1
115,5		129,4	151
363			154
192			78,9
197			202,5
199			168
242			199,5
298			27,1
301			150,9
342			
