# Supplementary material for: Alterations in innate immune responses of patients with chronic rhinosinusitis related to cystic fibrosis
Source: PLoS One. 2022 May 6;17(5):e0267986. doi: 10.1371/journal.pone.0267986 (PMC9075614; doi:10.1371/journal.pone.0267986)
Supplement: S3 File — (PDF) [file pone.0267986.s005.pdf]

Ânion superoxide			
Baseline			
Control	Cystic fibrosis	CF+CRS+NP+	CF+CRS+NP-
39	20	19,5	27,4
33	51	65	38,9
87,5	45,5	15	69,5
84,5	11,5	45	22
81,5	16	43,5	77
89			30
90,5			
80			
96,5			
76,5			
80			
94			
93,5			
66			
Without reduction/without fag			
Control	Cystic fibrosis	CF+CRS+NP+	CF+CRS+NP-
10,7	57,5	75	76,5
4	35	51,5	25
0,5	51	41	15,5
0,5	28,1	16,1	16
0,1	8,5	33	26
4,5			18,5
3			
0,2			
1,5			
4			
1,5			
5			
0,5			
1,5			
Without reduction/with fag			
Control	Cystic fibrosis	CF+CRS+NP+	CF+CRS+NP-
4,5	26,5	17	9
2	31,5	36	61
0,5	13	7	9
0,1	53,6	23,4	46
0,5	70,5	21,5	4
0,11			52
0,21			
3,5			

0,09			
0,13			
0,13			
0,15			
0,14			
With reduction/without fag			
Control	Cystic fibrosis	CF+CRS+NP+	CF+CRS+NP-
27	10,5	7,5	14,5
18,5	31	14	6
26,5	34,5	52	64
23	8,7	57,1	19
23	9,5	28,5	69
14		40	14
31,5			
11,5			
23,5			
26,5			
16,5			
30			
26,5			
10,5			
With reduction/with fag			
Control	Cystic fibrosis	CF+CRS+NP+	CF+CRS+NP-
55	5,5	0,5	0,2
71	2,5	1	8
76	1,5	0,1	11,5
77	9,7	3,4	19
81	10,5	9	1
65,5			15,5
88,5			
71,5			
69,5			
82			
63,5			
73			
88			
