# Supplementary material for: Alterations in innate immune responses of patients with chronic rhinosinusitis related to cystic fibrosis
Source: PLoS One. 2022 May 6;17(5):e0267986. doi: 10.1371/journal.pone.0267986 (PMC9075614; doi:10.1371/journal.pone.0267986)
Supplement: Attachment — Submitted filename: Reviewer Comments.docx [file pone.0267986.s006.docx]

Reviewer Comments:

The article by Gustavo Lara Rezende and colleagues, “Alterations in innate immune responses of patients with chronic rhinosinusitis related to cystic fibrosis”, offers insights on the role of monocytes and neutrophils in CF +/- CRS+/-NP and importance for the need of more diagnostic and treatments options for this illness. Overall, the study has been well-executed, and the manuscript well written providing explanations and background information to make the manuscript an enjoyable and easy to understand read.

I have minor suggestions to improve the manuscript.

1. **Materials and Methods**

Formatting:

- Pg.9 – ‘In addition, endoscopic signs… meatus or were necessary to define CRS’. The word “OR” should be deleted.
- Pg. 10 – Second paragraph… ‘Saccharomyces cerevisiae yeast are phagocytosed via receptors. Phagocytosis…. Align the paragraph accordingly.

2. **Table 1:** As transplant patients are given immunosuppressive (IS) drugs, it would be nice to mention the # of years post-transplant for the specific subject under the CF+CRS with NP group and clearly specify whether this subject was still on any IS drugs or not to infer that any alterations in this subject’s immune system was not contributed from post-transplant treatment.
3. **Figures 1 and 2:**

- It would be better to rename A, B and C as top, middle and bottom panel respectively to avoid confusion with the figure names A1, A2, etc. and as “A, B and C” are not stated on the figures.
- Figure 1A3- under 20 yeasts/neutrophils there is an additional undefined box plot along with a statistical line after CF+CRS without NP. Please clarify.

4. **Supplementary Table 1 & 2:** Kindly explain what you mean by ‘*values different from their respective control’?
5. **Supplementary Table 2:** Both CF+CRS with/without NP have the same number of yeasts ingested by monocytes 2.1 (20 years per cell). Yet a statistical difference is only observed for Ctrl vs CF+CRS with NP and not Ctrl vs CF+CRS without NP. Could you provide some insights on the same?
